# Stratified distribution of Th17 and Treg cells in patients with multi-stage rheumatoid arthritis

**DOI:** 10.1186/s13075-023-03041-7

**Published:** 2023-04-04

**Authors:** Rui Su, Baochen Li, Ruihe Wu, Yuhuan Xie, Anqi Gao, Chong Gao, Xiaofeng Li, Caihong Wang

**Affiliations:** 1 Department of Rheumatology, the Second Hospital of Shanxi Medical University, Taiyuan, Shanxi China; 2 Shanxi Key Laboratory of Immunomicroecology, Taiyuan, Shanxi China; 3 Brigham and Women’s Hospital/Children’s Hospital Boston, Joint Program in Transfusion Medicine, Harvard Medical School, PathologyBoston, USA

**Keywords:** Early rheumatoid arthritis, Regulatory T cells, T-helper 17 cells, Heterogeneity

## Abstract

**Objective:**

Rheumatoid arthritis (RA) is a typical, progressive autoimmune disease. Its occurrence and development are associated with dysregulation of T and B cell numbers. However, the specific immune characteristics of different RA courses remain incompletely defined. Here, we describe the peripheral blood lymphocyte subsets, particularly CD4 + T subsets, of different RA courses with a focus on early RA (Ea-RA).

**Methods:**

In all, 131 patients with Ea-RA, 117 with advanced RA (Ad-RA), and 109 with treated RA (Tr-RA) were enrolled. We collected general clinical data. Whole blood samples obtained from the patients and 97 healthy controls (HCs) were analysed via flow cytometry.

**Results:**

Decreased absolute NK cell numbers and increased CD4/CD8 T cell ratios were observed in different RA groups, including Ea-RA, compared to healthy controls. In Ea-RA patients, the Th17 and Treg cell numbers were similar to those in HCs. We performed k-means clustering based on the profiles of Th17 and Treg cells for patients with multi-stage of RA. We identified three patient types: type A characterised by relatively low Treg and Th17 cell numbers, type B with moderate levels of Treg cells and levels of Th17 cells similar to that of type C patients, and type C with high levels of Treg cells and levels of Th17 cells similar to that of type B patients.

**Conclusion:**

The immune characteristics of Ea-RA patients differ from those of HCs; an immune system disorder is apparent although no differences in Th17 and Treg levels were evident between Ea-RA patients and HCs. We found distributional heterogeneities of Th17 and Treg cells in patients with multi-stage of RA. Stratified management based on such heterogeneity may serve as a useful novel immunotherapy allowing of early intervention.

## Introduction

Rheumatoid arthritis (RA) is a typical autoimmune disease featuring synovial inflammation that triggers cartilage and bone destruction. There are three key phases: breakdown of systemic autoimmune tolerance, a transition from an asymptomatic autoimmune stage to tissue inflammation, and a transition from acute synovitis to chronic persistent inflammation [1, 2]. The breakdown of immune tolerance, immune system-mediated tissue damage, and autoantibody development usually precede clinical disease by years or even decades. The destruction of immune tolerance triggers autoantibody generation; this is a key feature of RA occurrence and development. CD4 + T subsets disorder is involved in RA pathogenesis. After the discovery of the classical Th1/Th2 imbalance, breakthrough studies on the Th17 /Treg imbalance followed. Treg cells play a vital role in the maintenance of immunological self-tolerance and homeostasis; they suppress the overactivation of effector CD4 + T cells [3, 4]. Th17 cells and Treg cells play key roles in the maintenance of immune homeostasis; an early recovery of such homeostasis is crucial when treating early RA (Ea-RA). Treat-to-target strategies can achieve drug-free remission in Ea-RA patients; if this is lacking, irreversible disease progression is likely. There is thus a window of opportunity for RA treatment [5]. Stratification of RA patients, particularly those with undifferentiated arthritis, is urgently needed for the delivery of precision medicine [6]. Stratification based on immune system characteristics is very attractive. The current data on Treg cell numbers and activities at different stages of RA are both contradictory and unclear [7–9]. Heterogeneity may be in play.

In this study, we retrospectively analysed the lymphocyte subsets of peripheral blood, particularly the CD4 + T subsets, in patients with different courses of RA. We performed k-means clustering based on the Th17 and Treg cell profiles. We thus explored heterogeneities in cell numbers and proportions throughout the course of RA in an effort to develop new early interventions and precise individualised treatments.

## Materials and methods

### Subjects

In all, 131 patients with Ea-RA, 117 with advanced RA (Ad-RA), and 109 with treated RA (Tr-RA) were enrolled. All patients were hospitalised in between July 2017 and December 2021 in the Department of Rheumatology, the Second Hospital of Shanxi Medical University. Ea-RA patients met the 2010 RA diagnostic criteria, but disease duration was less than 1 year. Ad-RA patients met the ACR 1987 or 2010 criteria and the disease course exceeded 1 year. Neither of these groups had ever received glucocorticoids or disease-modifying antirheumatic drugs (DMARDs). Tr-RA patients had received at least one treatment and had been re-admitted with relapses, 20 of them were taking prednisolone (≤ 10 mg/per day) before blood samples were taken, 39 were on DMARDs, and the remainder had stopped drug treatment more than 3 months prior. The medications are summarised in Table 1. Patients with tumours or severe infections, and pregnant women, were excluded. Blood samples were obtained from all subjects prior to treatment initiation. We also recruited 97 healthy controls (HCs) age- and sex-matched to RA patients. The different RA groups were similar in terms of sex and age distributions. The study was approved by the ethics committee of the Second Hospital of Shanxi Medical University (approval no. [2019] YX No. 148 [105]).

**Table 1:** Demographic and clinical characteristics of the study subjects

Characteristics	Ea-RA (*n* = 131)	Ad-RA (*n* = 117)	Tr-RA (*n* = 109)	Healthy controls (*n* = 97)
Female, *n* (%)	82 (62.60%)	82 (70.08%)	81 (74.31%)	67 (69.07%)
Age, years	55.12 ± 15.34	56.59 ± 12.97	57.80 ± 12.13	51.30 ± 8.78
WBC (10^9^/L)	6.93 ± 2.13 a*	6.78 ± 2.19	6.89 ± 2.58 a**	6.16 ± 1.86
Hb (g/L)	117.54 ± 18.64 a*	123.41 ± 33.23	116.49 ± 24.30 a*	126.37 ± 11.56
PLT (10^9^/L)	266.50 ± 107.03 a***	289.47 ± 106.63 a***	278.29 ± 98.93 a***	190.59 ± 42.07
ESR (mm/h)	49.74 ± 33.21	52.00 ± 36.00	57.63 ± 37.15	_
DAS-28-ESR	4.81 ± 3.02	4.49 ± 1.36	4.74 ± 1.28	_
RF (U/mL)	178.11 ± 306.95	203.57 ± 284.71	225.75 ± 371.70	_
Anti-CCP (U/mL)	498.35 ± 486.01	604.92 ± 586.05	651.99 ± 550.60	_
Anti-MCV (U/mL)	422.50 ± 388.21	418.83 ± 392.01	412.27 ± 388.11	_
ANA *n* (%)	60 (45.8%)	67 (57.3%)	57 (52.3%)	_
Pred (≤ 10 mg/day	-	-	20/109	_
DMARDs	-	-	39/109	

*WBC* white blood cells, *Hb* haemoglobin, *PLT* platelets, *ESR* erythrocyte sedimentation rate, *CRP* C-reactive protein, *DAS28* Disease Activity Score 28, *RF* rheumatoid factor, *Anti-CCP* anti-cyclic citrullinated peptide antibody, *Anti-MCV* anti-mutated citrullinated vimentin, *ANA* antinuclear antibodies, *DMARDs* disease-modifying antirheumatic drugs

^*^ *p* < 0.05

^**^ *p* < 0.01

^***^ *p* < 0.001

### Clinical and laboratory data collection

Clinical and laboratory parameters were retrospectively collected from clinical databases. General clinical data included age and sex. Serological parameters included routine blood counts, erythrocyte sedimentation rates (ESR), and the levels of rheumatoid factor (RF), anti-cyclic citrullinated peptide antibody (anti-CCP), and anti-mutated citrullinated vimentin (anti-MCV). Disease activity was assessed using the Disease Activity Score 28 joint count (DAS28)-ESR instrument.

### Flow cytometry

Phenotyping of peripheral blood lymphocytes (T/B/NK/CD4^+^/CD8^+^T cells): 50 μL amounts of blood with an anticoagulant were added to both Trucount A and B tubes; 20 μL CD3 − FITC/CD8 − PE/CD45 − PerpCP/CD4 − APC was added to the A tube and 20 μL CD3 − FITC/CD16 + CD56 − PE/CD45 − PerpCP/CD19 − APC was added to the B tube, followed by incubation for 20 min in the dark. Then, 450 μL hemolysin was added to each tube, with gentle swirling. After standing at room temperature for 15 min, flow cytometry proceeded. The percentages and absolute counts of CD3^+^CD19^−^T cells, CD3^−^CD19^+^ B cells, CD3^+^CD4^+^ T cells, CD3^+^CD8^+^ T cells, and CD3^−^CD16^+^CD56^+^ NK cells were automatically calculated using BD Multitest software (BD Biosciences).

**Fig. 1:**
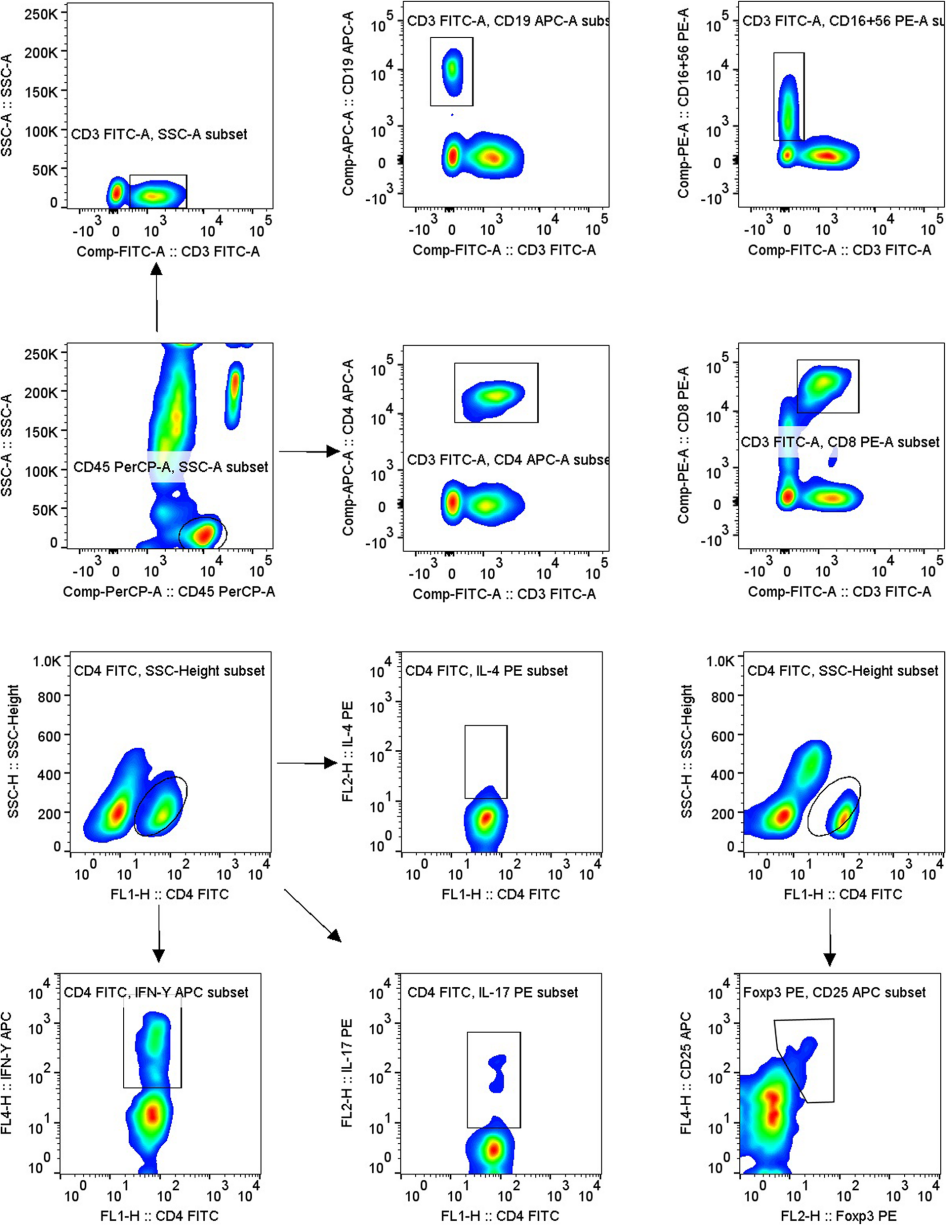
A Schematic of the gating for flow cytometric analysis of lymphocyte subsets. Total T cells: CD3 + CD19 − , B cells: CD3 − CD19 + , NK cells: CD3 + CD16 + CD56 + , CD4 + T cells: CD3 + CD4 + , CD8 + T cells: CD3 + CD8 + , Th1 cells: CD4 + IFNγ + , Th2 cells: CD4 + IL − 4 + , Th17 cells: CD4 + IL − 17 + , Treg cells: CD4 + CD25 + Foxp3 + . B Proportions and absolute numbers of T, B, and NK cells in different groups. Absolute numbers and percentages of CD4 and CD8 cells, and the ratios thereof, in HCs and patients with Ea-RA, Ad-RA, and Tr-RA. Ea-RA: Early RA; Ad-RA: Advanced RA; Tr-RA: Treated RA; HC: Healthy controls. (*p < 0.05, **p < 0.01, ***p < 0.001)

CD4 + T subsets detection (Th1/Th2/T17/Treg cells): 80 μL blood with heparin (an anticoagulant) was mixed with 10 μL PMA, 10 μL ionomycin, and 1 μL Golgi Stop, followed by incubation for 5 h at 37 °C. The samples were divided into two tubes, both of which received anti-CD4-FITC, followed by incubation for 30 min in the dark. A fixation/permeabilization mixture was added, followed by incubation for 30 min in the dark. Anti-IFN-APC and anti-IL-4-PE were added to one tube to stain Th1/Th2 cells. Anti-IL-17-PE was added to the other tube to stain Th17 cells. After 30 min in the dark, the cells were washed with PBS and detection proceeded. To detect Treg cells, 80 μL anticoagulant was added to the tube with anti-CD4-FITC and anti-CD25-APC and after 30 min in the dark, 1 mL fixation/permeabilization mixture further added, followed by incubation for 30 min in the dark. To detect Treg cells, anti-Foxp3-PE was added to blood followed by incubation for 30 min in the dark. All samples were analysed within 24 h of preparation, and the absolute counts of the CD4 + T subsets were the percentages of all subsets of CD4 + T cells (cells/μL) (Fig. 1A).

### Statistical analysis

SPSS ver. 20.0 and GraphPad Prism ver. 8.0 were used to derive descriptive and inferential statistics. Between-group differences in continuous variables were analysed using the independent samples *t*-test for normally distributed data and the Mann–Whitney *U*-test for non-normally distributed data. Categorical variables were compared using the chi-square test. Correction for multiple comparisons was conducted using the Benjamini–Hochberg false discovery rate (FDR). A *p*-value < 0.05 (two-sided) was considered statistically significant. Clustering of subjects by the Th17 and Treg cell numbers employed the k-means method.

## Results

### Higher levels of white blood cells and platelets, and lower haemoglobin levels, in RA patients

Ea-RA and Tr-RA patients exhibited higher white blood cell (WBC) counts and lower haemoglobin (Hb) levels than HCs. The platelet (PLT) counts of all RA patients were higher than that of HCs. There were no significant differences in ESR, DAS28, or levels of RF, anti-CCP, or anti-MCV among the RA groups. The antinuclear antibody (ANA) positivity rate was 57–67% among RA patients but did not differ by RA stage (Table 1).

### Reduced NK and T cell numbers in Ea-RA patients

We explored T, B, and NK cells in each group. The absolute numbers of T cells and also NK cells were lower in Ea-RA, Ad-RA, and Tr-RA patients than in HCs. However, there were no significant differences in either the absolute number or proportion of B cells among the three RA groups and HCs (Fig. 1).

### Increased CD4 + /CD8 + T cell ratios in the RA groups

**Fig. 2:**
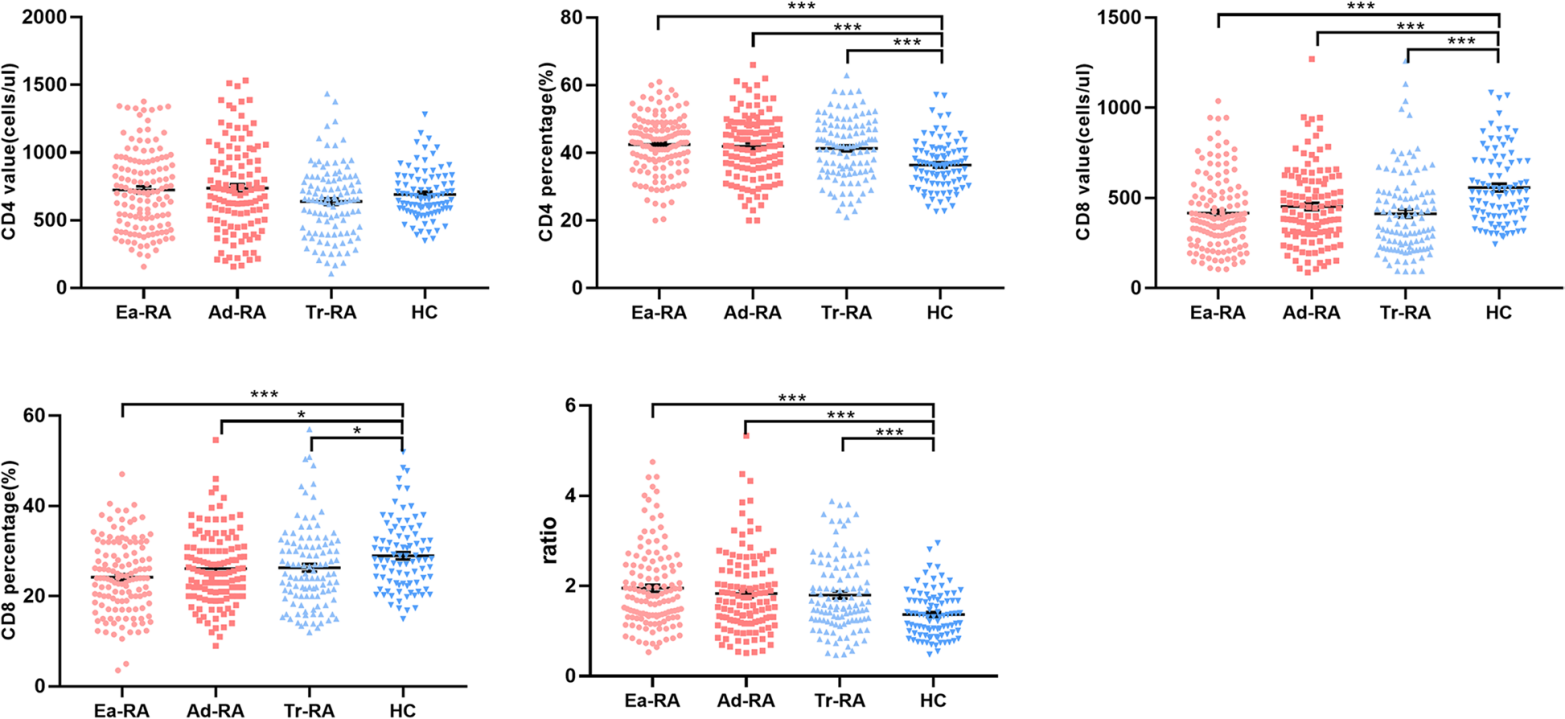
Proportions of CD4 and CD8 cells and the CD4/CD8 ratios in the various RA groups and HCs. Absolute numbers and percentages of CD4 and CD8 cells, and the ratios thereof, in HCs and patients with Ea-RA, Ad-RA, and Tr-RA. (*p < 0.05, **p < 0.01, ***p < 0.001)

We sought differences in the proportions and absolute numbers of CD4 + and CD8 + T cells among the different groups. All RA groups evidenced increased CD4 + T and decreased CD8 + T proportions compared to HCs, and thus elevated CD4/CD8 ratios. The absolute numbers of CD8 + T cells in all RA groups were lower than in HCs, but the absolute numbers of CD4 + T cells in all RA groups and HCs were similar (Fig. 2).

### Th17/Treg imbalances in patients with Ad-RA and decreased Treg cell numbers in Tr-RA patients compared to HCs

**Fig. 3:**
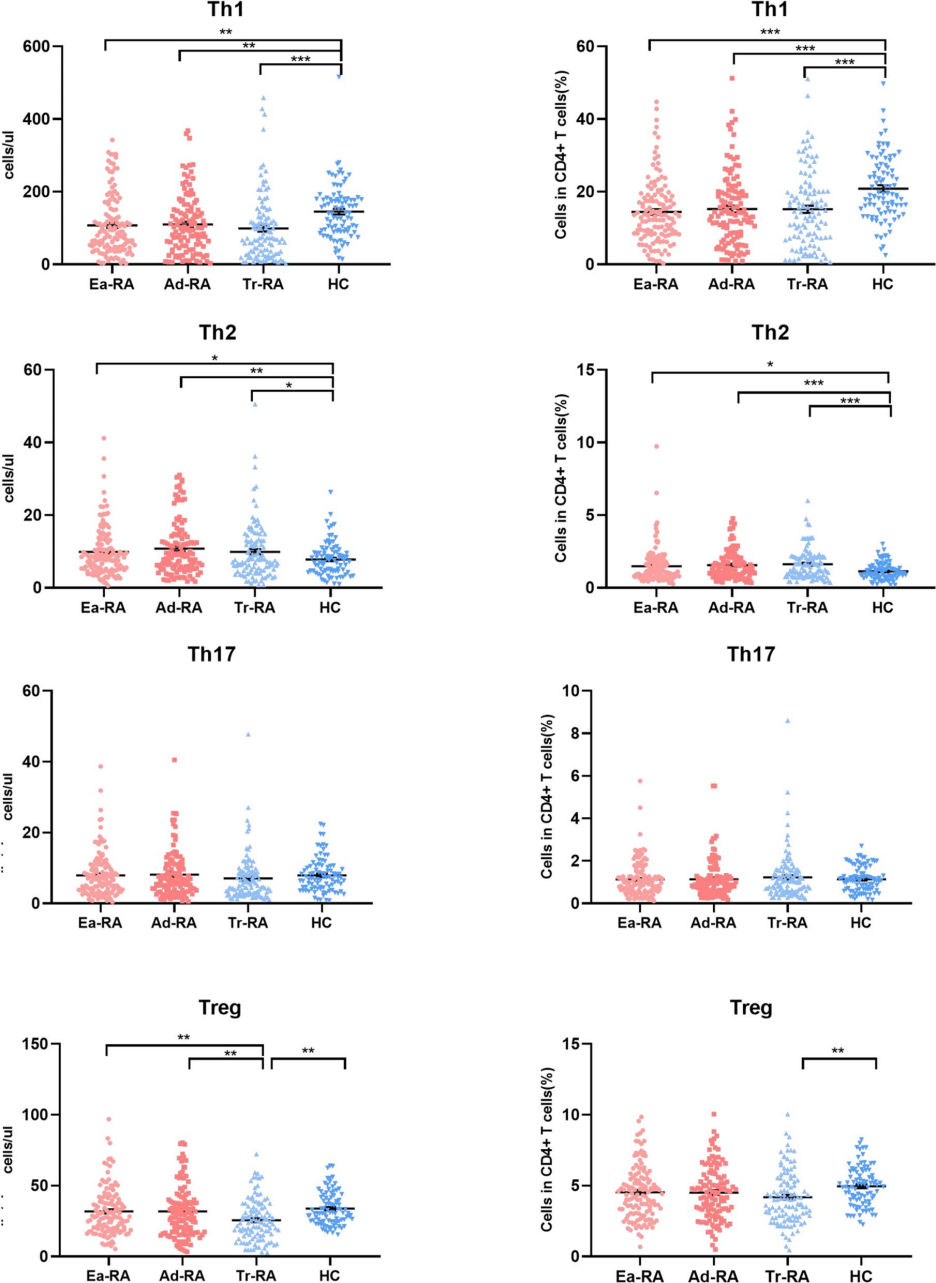
CD4 subset numbers in the various RA groups and HCs. Absolute numbers and percentages of Th1, Th2, Th17, and Treg cells in HCs and patients with Ea-RA, Ad-RA, and Tr-RA (*p < 0.05, **p < 0.01, ***p < 0.001)

**Fig. 4:**
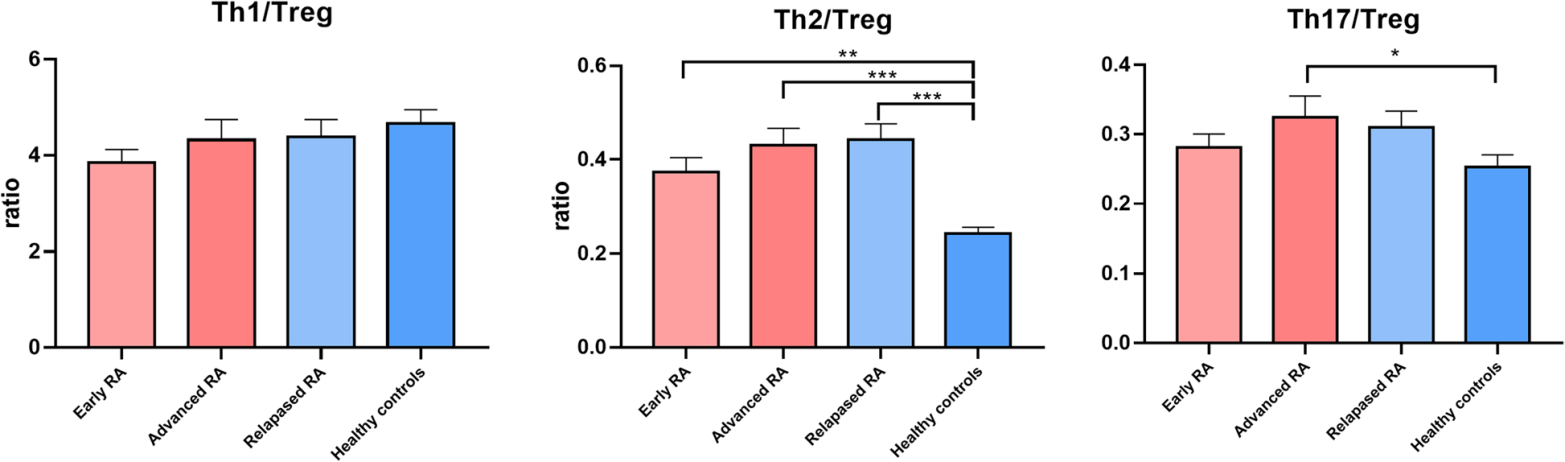
The ratios of effector T (Th1, Th2, and Th17) and Treg cells to all immune cells in the Ea-RA, Ad-RA, and Tr-RA groups; and HCs. (*p < 0.05, **p < 0.01, ***p < 0.001)

Certain CD4 + T subsets are associated with RA. We analysed known CD4 + T subsets, thus Th1, Th2, Th17, and Treg cells. Ea-RA patients evidenced decreased absolute numbers and proportions of Th1 cells but increased numbers and proportions of Th2 cells. However, the Th17 cell numbers did not significantly differ among the RA groups. Particularly, the absolute number of Treg cells of Ea-RA patients was similar to that of HCs but higher than that of Tr-RA patients. However, the absolute number and proportion of Treg cells in Tr-RA patients were less than in HCs. We further analysed the Th1/Treg, Th2/Treg, and Th17/Treg ratios in Ea-RA patients. The Th2/Treg ratio was elevated compared to that of HCs, as was the case for Ad-RA and Tr-RA patients. The Th1/Treg ratios did not differ among the RA groups. The Th17/Treg ratio of Ad-RA patients was higher than that of HCs (Figs. 3 and 4).

### Distributional heterogeneities of Th17 and Treg cell numbers in Ea-RA patients

**Fig. 5:**
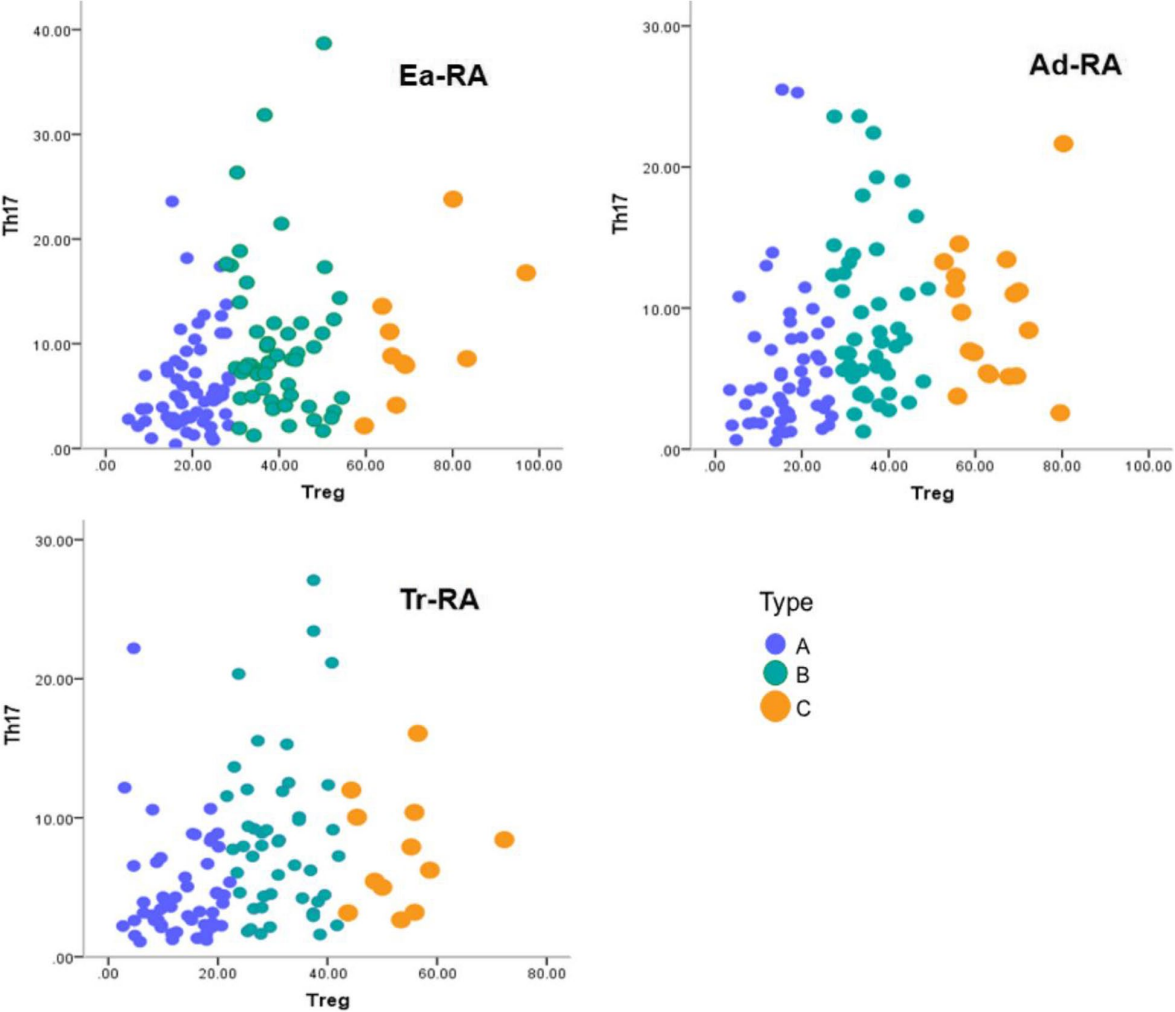
Scatter diagram of k-means clustering based on Th17 and Treg cell numbers in Ea-RA, Ad-RA, and Tr-RA patients. Ea-RA: Early RA (*n* = 131), Ad-RA: Advanced RA (*n* = 117), Tr-RA: Treated RA (*n* = 109). Type A: Low levels of Th17 and Treg cells. Type B: Moderate levels of Treg cells and levels of Th17 cells similar to that of Type C patients. Type C: High levels of Treg cells and levels of Th17 cells similar to that of Type B patients

It is very surprising that the levels of Th17 and Treg cells in Ea-RA patients were similar to those of HCs. Recent studies have indicated that patient stratification is essential when managing Ea- and pre-RA. We used k-means clustering to classify Ea-RA patients by their Th17 and Treg cell profiles. This identified three types of patients: type A (*n* = 66) characterised by relatively low Treg and Th17 cell numbers, type B (*n* = 55) with moderate levels of Treg cells, and type C (*n* = 10) with higher levels of Treg cells. The Th17 cell level of type B was similar to that of type C, suggesting that most Ea-RA patients are of types A and B. In fact, the same trend was observed in Ad-RA and Tr-RA patients (*p* > 0.05) but the overall numbers of Th17 and Treg cells were lower (Fig. 5).

## Discussion

RA development may be halted or even prevented by very early treatment during a therapeutic window of opportunity or an appropriate pre-disease stage. Longitudinal studies on RA patients at different disease stages are essential in the present era of precision medicine [10]. Hence, identification of the lymphocyte immunophenotype in early stages of RA aids an in-depth understanding of the immune mechanism in play at that time, and the selection of appropriate treatment strategies.

We found that the PLT levels at all stages of RA were higher than in HCs. Recent studies have found that PLTs regulate haemostasis and thrombosis, infection, and innate and adaptive immunity [11]. In a previous study, the PLT counts of RA patients were significantly higher than those of HCs [12]. Our results are consistent with this; specifically, we included patients with RA of various stages. Notably, we found that 57–67% of RA patients of all stages were ANA-positive. Other recent studies have reported positivity rates of 35–60% [13, 14]. However, ANA positivity in RA patients has not been extensively studied. We found that the rate was higher in patients with all stages of RA than in HCs. The clinical features, and diagnostic and prognostic utilities, of ANA positivity in RA patients deserve further exploration.

An immune system disorder contributes to RA pathogenesis. Further study of lymphocyte subsets that are active during Ea-RA may allow treatment that achieves remission, perhaps even without drugs. Our results suggest that the overall T, B, NK, CD4 + , and CD8 + T cell characteristics of Ea-RA patients do not differ from those at later stages of RA. NK cells participate in RA immunopathogenesis, but their numbers and functions remain somewhat unclear. Chalan et al. found that total NK cell numbers were lower in patients with seropositive RA [15]. Our results are consistent with this; decreased absolute numbers of NK cells were observed in all RA groups including Ea-RA. However, other studies have reported elevated or similar numbers of NK cells in RA patients compared to HCs [16, 17]. Further exploration of the levels and functions of the different RA NK subsets is necessary. In addition, increased CD4 + and decreased CD8 + T cell numbers, and thus increased CD4/CD8 ratios, were observed in different RA groups including Ea-RA. However, compared to HCs, the absolute numbers of T cells in Ea-RA and Tr-RA patients decreased. These data indicate that Ea-RA and HC subjects differ; an immune disorder has clearly occurred in the patients.

B cells play key roles in antigen presentation, cytokine secretion, and production of the autoantibodies typical of RA. However, we found that the B cell numbers did not significantly differ between patients and HCs. This is not necessarily surprising. B cells are very complex. We did not distinguish pathogenic from protective B cells. Therapeutic strategies targeting certain B cells and B cell checkpoints are promising. Accurate classification and a better understanding of the functions of B cell subsets are also important [18].

Changes in the function and distribution of CD4 + T subsets play crucial roles in RA. However, the distributional characteristics of CD4 + subsets at different stages of RA, particularly Ea-RA, have been but little explored. It is generally accepted that a Th1/Th2 imbalance attributable to increased levels of pro-inflammatory Th1 cells and decreased levels of Th2 cells contribute to RA pathogenesis. However, certain newly discovered CD4 + T subsets challenge the classical Th1/Th2 paradigm. We found decreased absolute numbers and proportions of Th1 cells but increased numbers and proportions of Th2 cells in different RA groups including Ea-RA. This is consistent with a previous study that examined the T cell subsets of untreated Ea-RA patients and HCs and found that the patients evidenced more Th2 but fewer Th1 cells than HCs [19]. A standard flow cytometry gating strategy and efforts to ensure patient homogeneity may help resolve the contradiction.

Many studies support the viewpoint that Th17 /Treg imbalance is the pathogenic mechanism of RA. It is generally agreed that the levels of Th17 cells and IL-17 in patients with established RA are higher than in healthy controls. However, many studies have included subjects with different disease durations; some have been newly diagnosed Ea-RA patients and some have had established RA. We found that the absolute numbers and percentages of Th17 cells in Ea-RA patients did not differ from those of HCs. On the contrary, others have reported either increased or reduced Th17 percentages in Ea-RA patients compared to HCs [20–22]. The absolute numbers of Treg cells in Ea-RA and Ad-RA patients were higher than in Tr-RA patients but similar to those of HCs. However, the Th17 and Treg cell numbers were higher than those of HCs in only Ad-RA patients. Thus, Th17 and Treg cell numbers are relatively stable in the early stages of RA; immune homeostasis is not completely destroyed. The breakdown of self-immune tolerance caused by the dysregulation of Treg cells is a key mechanism of RA progression [23]. The increased Treg numbers in Ea-RA and Ad-RA patients compared to Tr-RA patients suggest that, on the one hand, Treg cells remain relatively normal in the early stages of disease, and on the other, that treatment reduces Treg cell numbers. However, one study found that both the percentage and absolute number of Treg cells in untreated Ea-RA patients were lower than in HCs [7]. Therefore, the data are contradictory. The reason must be further explored.

It is very surprising that the levels of Th17 and Treg cells in Ea-RA patients are similar to those of HCs. To explore possible heterogeneities in the distributions of Th17 and Treg cells in RA patients, we performed k-means clustering based on the profiles of such cells in Ea-RA patients. We identified three types of such patients: type A characterised by relatively low Treg and Th17 cell levels, type B with moderate levels of Treg cells, and type C with higher levels of Treg cells. In fact, the level of Th17 cells in type B patients was similar to that in type C patients. Thus, the distributions of Th17 and Treg cells were heterogeneous in Ea-RA patients, most of whom were of types A and B. A small number of Ea-RA patients evidenced high Treg levels. Thus, stratified management is appropriate even for pre-RA patients. In the early stages of the disease, precise stratification by the extent of immune system heterogeneity would improve outcomes. A 25-year longitudinal cohort study emphasised that patients with undifferentiated arthritis did not benefit from enhanced DMARD strategies; stratification methods were required [6]. The trends in Ad-RA and Tr-RA patients were similar. However, the overall levels of Th17 and Treg cells were lower in such patients, perhaps reflecting disease progression or treatment. These results also suggest that Th17 and Treg cell heterogeneities were evident at different stages of RA. Although the numbers of Th17 and Treg cells differed, the overall distribution trends were similar. Unlike traditional strategies, the current therapeutic approaches seek to reinstate immunological self-tolerance; such therapies exhibit great potential [24]. Therefore, it is essential to further explore whether patient stratification based on Th17 and Treg cell numbers can improve the clinical management of different stages of RA.

Given the individual differences among Ea-RA patients, it is difficult to define specific treatment strategies. Personalised decisions based on specific biological markers are required. For example, one report found that RA patients with high levels of Treg CTLA4 expression responded better than others to MTX. It is important to individualise drug selection and prediction of efficacy based on the Th17 and Treg cell levels [7]. Although Th17 and Treg heterogeneities were apparent in patients with RA of different stages, we have not yet assessed whether the different RA classes differed in terms of treatment response and prognosis. Follow-up studies are needed.

## Conclusion

The distributions of Th17 and Treg cells in patients with multi-stage of RA are heterogeneous. Stratified management based on such heterogeneity would be beneficial. Further exploration of Th17 and Treg status in Ea-RA or pre-RA patients may indicate new strategies allowing early drug-free RA remission.

## Data Availability

All data generated or analysed in this study are available from the corresponding author upon reasonable request.

## Abbreviations

RA: Rheumatoid arthritis
Ea-RA: Early RA
Ad-RA: Advanced RA
Tr-RA: Treated RA
HCs: Healthy controls
DMARDs: Disease-modifying antirheumatic drugs
ESR: Erythrocyte sedimentation rates
RF: Rheumatoid factor
Anti-CCP: Anti-cyclic citrullinated peptide antibody
Anti-MCV: Anti-mutated citrullinated vimentin
WBC: White blood cell
ANA: Antinuclear antibody
Hb: Haemoglobin
PLT: Platelet
DAS28: Disease Activity Score 28 joint count
